# E3 Ubiquitin Ligase RNF13 Suppresses TLR Lysosomal Degradation by Promoting LAMP‐1 Proteasomal Degradation

**DOI:** 10.1002/advs.202309560

**Published:** 2024-06-21

**Authors:** Wei Liu, Yuyang Wang, Shuo Liu, Xuan Zhang, Xuetao Cao, Minghong Jiang

**Affiliations:** ^1^ Department of Immunology Center for Immunotherapy Institute of Basic Medical Sciences Peking Union Medical College Chinese Academy of Medical Sciences Beijing 100005 China; ^2^ Department of Rheumatology Beijing Hospital National Center of Gerontology Institute of Geriatric Medicine Chinese Academy of Medical Sciences Beijing 100730 China

**Keywords:** endo‐lysosome, LAMP‐1, macrophages, RNF13, TLRs, ubiquitination

## Abstract

As a highly organized system, endo‐lysosomes play a crucial role in maintaining immune homeostasis. However, the mechanisms involved in regulating endo‐lysosome progression and subsequent inflammatory responses are not fully understood. By screening 103 E3 ubiquitin ligases in regulating endo‐lysosomal acidification, it is discovered that lysosomal RNF13 inhibits lysosome maturation and promotes inflammatory responses mediated by endosomal Toll‐like receptors (TLRs) in macrophages. Mechanistically, RNF13 mediates K48‐linked polyubiquitination of LAMP‐1 at residue K128 for proteasomal degradation. Upon TLRs activation, LAMP‐1 promotes lysosomes maturation, which accelerates lysosomal degradation of TLRs and reduces TLR signaling in macrophages. Furthermore, peripheral blood mononuclear cells (PBMCs) from patients with rheumatoid arthritis (RA) show increased RNF13 levels and decreased LAMP‐1 expression. Accordingly, the immunosuppressive agent hydroxychloroquine (HCQ) can increase the polyubiquitination of RNF13. Taken together, the study establishes a linkage between proteasomal and lysosomal degradation mechanisms for the induction of appropriate innate immune response, and offers a promising approach for the treatment of inflammatory diseases by targeting intracellular TLRs.

## Graphical abstract

Liu et al. identify lysosomal E3 ubiquitin ligase RNF13 as a positive regulator of the TLR‐triggered innate response through high‐content screening. They provide mechanistic insights into the interplay between lysosomal progression, inflammation and ubiquitylation, and highlight the important role of E3 ubiquitin ligases in the regulation of inflammatory responses and related autoimmune diseases.

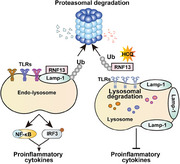

## 1 Introduction

Toll‐like receptors (TLRs) are a conserved family of pattern recognition receptors (PRRs) that are the first line of host defense against damage from external pathogens by recognizing pathogen‐associated molecular patterns (PAMPs).^[^ ^1^ ^]^ TLRs can also recognize internal damage‐associated molecular patterns (DAMPs), leading to the induction of inflammatory responses.^[^ ^2^ ^]^ Full activation of TLRs is required for the host to eliminate invading pathogens; however, inappropriate activation or excessive stimulation of TLRs can lead to the initiation and progression of chronic inflammatory disorders, cancer, and autoimmune diseases.^[^ ^2^, ^3^ ^]^ Thus, tight and precise regulation of TLR signaling pathways is critical for maintaining the balance between the activation and inhibition of TLR signaling in immune defense and disease pathogenesis.

TLRs are type I transmembrane proteins consisting of a ligand‐binding domain and an intracellular toll/interleukin 1 (IL‐1) receptor (TIR) homology domain.^[^ ^4^ ^]^ Most TLRs (TLR1, 2, 4, 5, and 6) are expressed on the cell surface, where they have been shown to detect membrane components of microorganisms, such as lipids, lipoproteins, and proteins.^[^ ^1a^ ^]^ Among these surface TLRs, TLR4 can be internalized and translocated to Rab5‐ and EEA1‐positive early endosomes, where it interacts with TRAM and TRIF and initiates TRIF‐dependent signaling upon activation.^[^ ^5^ ^]^ The other subset of TLRs, including TLR3, TLR7/8, and TLR9, are localized within intracellular compartments, such as the endoplasmic reticulum (ER), endosome, or lysosome, which mainly specialize in microbial nucleic acid detection, and initiate downstream signaling, resulting in the secretion of inflammatory cytokines, type I interferon (IFN‐I), and chemokines.^[^ ^1a^ ^]^ Acidification is a crucial event in endosome maturation. Drugs such as bafilomycin A1 (Baf‐A1), which neutralize the pH of endosomes and lysosomes, block TLR signaling.^[^ ^6^ ^]^ In recent years, several endo‐lysosomal proteins, such as Lyst^[^ ^7^ ^]^ and Vps33B,^[^ ^8^ ^]^ have been reported to play critical roles in the kinetics of endo‐lysosomal maturation. Furthermore, we previously demonstrate that late endosome/lysosome‐localized Rab7b functions as a negative regulator of either TLR4 or TLR9 signaling in macrophages by enhancing trafficking of TLRs to the endosome/lysosome and promoting degradation of TLRs.^[^ ^9^ ^]^ However, the detailed mechanisms involved in the regulation of lysosomal acidification and biogenesis upon TLR activation remain to be fully elucidated.

Ubiquitination, a widespread post‐translational modification (PTM), plays a critical role in regulating the activity of functional proteins, and is therefore strongly associated with a variety of cellular processes, including protein degradation and trafficking.^[^ ^10^ ^]^ The ultimate conjugation of ubiquitin (Ub) to a substrate requires the sequential action of a Ub‐activating enzyme (E1), Ub‐conjugating enzyme (E2), and Ub ligase enzyme (E3).^[^ ^11^ ^]^ The RNF family, which contains an N‐terminal RING domain, is the largest E3 ubiquitin ligase family.^[^ ^12^ ^]^ Several RNF family members localized in endosomal or lysosomal membranes, such as ZNRF1, ZNRF2, RNF152, RNF167, and RNF13, undergo extensive proteolysis in a proteasome‐dependent manner.^[^ ^13^ ^]^ The lysosomal RNF13 protein has been associated with tumorigenesis, ER stress, and myogenesis.^[^ ^13^, ^14^ ^]^ The C‐terminal of the RNF13 protein contains a RING domain that can potentially ubiquitinate substrates in organelles of the biosynthetic pathway,^[^ ^15^ ^]^ but the substrates of this lysosomal ubiquitin ligase have not yet been identified.

Here, we carried out high content screening (HCS) to investigate the function of the mouse RING domain E3 family in the regulation of endo‐lysosomal acidification, and identified that RNF13 promotes endosomal TLRs signaling by mediating K48‐linked polyubiquitination and proteasomal degradation of LAMP‐1. LAMP‐1 promotes acidification and the maturation of lysosomes, shortens TLRs retention in early endosome and increases translocation into the lysosomal compartment for degradation. Our study reveals a novel mechanism of TLR signaling regulation that combines proteasomes with lysosomes for the induction of optimal immune response, providing potential targets for control of inflammatory disease.

## 2 Results

### 2.1 RNF13 Suppresses Endo‐Lysosome Acidification and Promotes TLR‐Triggered Pro‐Inflammatory Cytokine Production in Macrophages

As cellular endo‐lysosome system plays an important role in regulating innate immunity, we screened the function of 103 mouse RING‐domain E3 ubiquitin ligases in regulating acidification of endo‐lysosome by high‐content screening (HCS). We transfected mouse primary peritoneal macrophages with small interfering RNAs (siRNAs) targeting candidate genes and then test the fluorescence intensity of pHrodo that represents the acidity of endo‐lysosome upon lipopolysaccharide (LPS) treatment. Among these E3 ligases, knockdown of lysosomal RNF13 robustly enhanced the acidification of endo‐lysosome (Figure S1A,B, Supporting Information), so we focused on RNF13 for further study.

To fully investigate the role of RNF13, we first analyzed the expression of RNF13 in different mouse primary immune cells and found that RNF13 was mainly expressed in macrophages (Figure S1C, Supporting Information). Notably, upon TLRs ligands LPS, Poly I: C and CpG ODN or *listeria monocytogenes* (*L. M*.) treatment, the protein level of RNF13 were gradually decreased in a time‐dependent manner (Figure S1D, Supporting Information), consistent with GEO profile (GDS4419, GDS2221), indicating that RNF13 may play important roles in innate immunity. Moreover, RNF13 displayed self‐ubiquitination activity as the expression of RNF13 could be notably increased upon MG132 treatment, suggesting that RNF13 is degraded by self‐ubiquitination (Figure S1E, Supporting Information).

**Figure 1:**
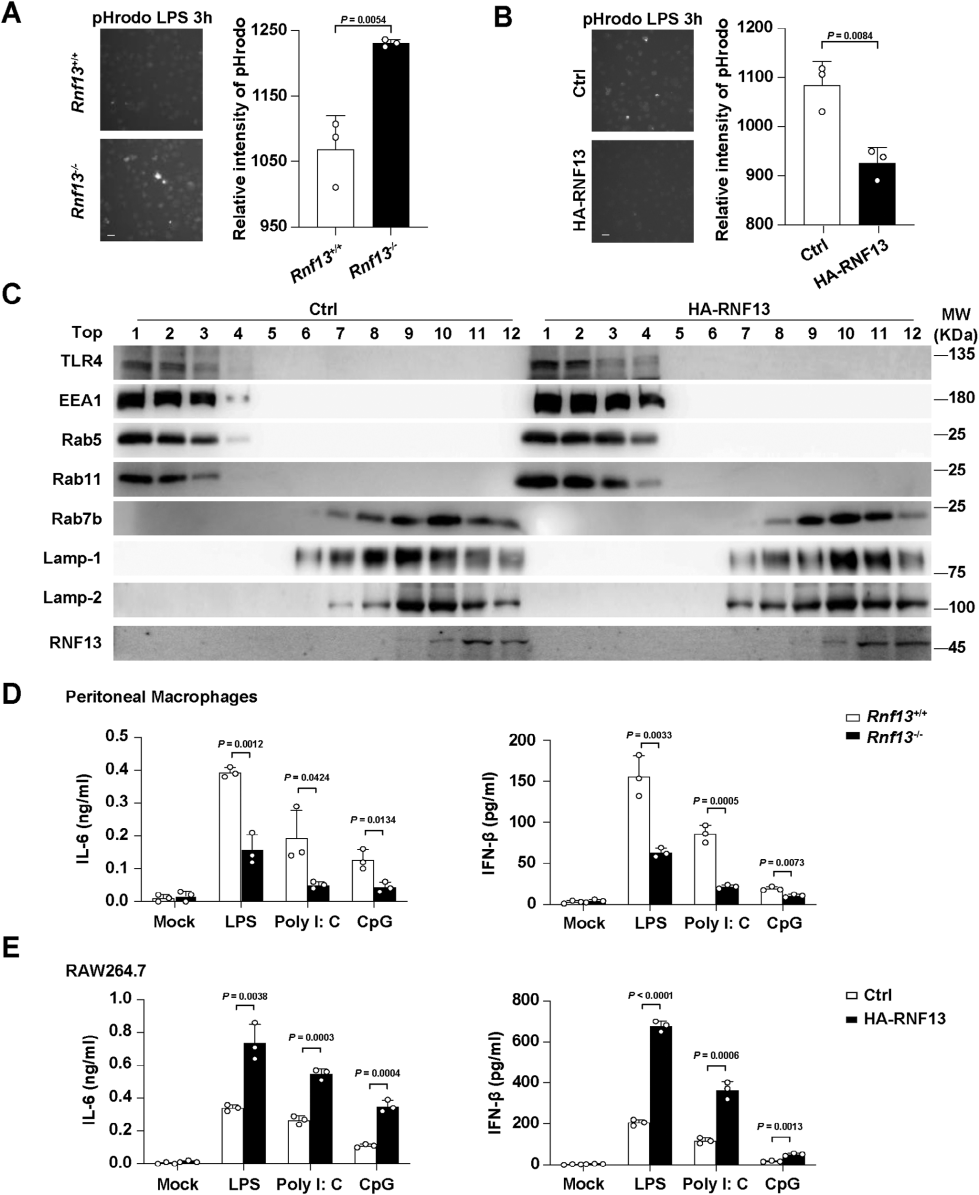
RNF13 suppresses endo‐lysosome acidification and promotes TLR‐triggered pro‐inflammatory cytokines production in macrophages. A) Images shot by HCS from pHrodo assay of endo‐lysosome acidification in *Rnf13* ^+/+^ or *Rnf13* ^−/−^ RAW264.7 cells along with LPS stimulation for 3 h. The relative intensity of pHrodo represents the acidity, which were from three independent experiments. (Scale Bar, 5 µm) B) Images shot by HCS from pHrodo assay of endo‐lysosome acidification in control (Ctrl) or RNF13 overexpressing (HA‐RNF13) RAW264.7 cells along with LPS stimulation for 3 h. The relative intensity of pHrodo represents the acidity, which were from three independent experiments. (Scale Bar, 5 µm.) C) Western blot assay of subcellular fraction in control (Ctrl) or RNF13 overexpressing (HA‐RNF13) RAW264.7 cells treated with LPS for 3 h. Top1 to Top12 orderly contains the supernatant of different subcellular fractions, respectively, referring to *Subcellular fraction assay*. D) ELISA assay of IL‐6 in the supernatants of primary peritoneal macrophages from *Rnf13* ^+/+^ or *Rnf13* ^−/−^ mice treated with LPS (100 ng mL^−1^), Poly I: C (10 µg mL^−1^) or CpG (2 µm) for 6 h, respectively. E) ELISA assay of IFN‐β in the supernatants of control (Ctrl) or RNF13 overexpressing (HA‐RNF13) RAW264.7 cells treated with LPS (100 ng mL^−1^), Poly I: C (10 µg mL^−1^) or CpG (2 µM) for 6 h, respectively. Data are representative of three independent experiments (A–C) or shown as mean ± SD of n = 3 biological replicates (A, B, D, E), two‐tailed unpaired Student's *t*‐test (A, B, D, E).

To confirm the effect of RNF13 in the production of proinflammatory cytokines, we generated RNF13‐deficient (*Rnf13* ^−/−^) RAW264.7 cells through the CRISPR‐Cas9 system (Figure S2A, Supporting Information) and RNF13 stable overexpressing (HA‐RNF13) RAW264.7 cells (Figure S2B, Supporting Information). 3 h after LPS treatment, the immunofluorescence intensity of pHrodo was robustly enhanced in *Rnf13* ^−/−^ cells (**Figure** **1**A) and significantly attenuated in RNF13‐overexpressing cells (Figure 1B), indicating that RNF13 inhibited endo‐lysosome acidification. Consistently, increased expression of RNF13, mainly detected in lysosome, resulted in increased expression of endosome‐localized proteins such as EEA1, Rab5, Rab11 and TLR4, and decreased expression of lysosome‐localized proteins such as Rab7b and LAMP‐1 (not including LAMP‐2) (Figure 1C). In addition, the subcellular fractions containing endosome‐localized proteins were expanded, whereas lysosome‐localized proteins were restricted in RNF13‐overexpressing cells (Figure 1C), indicating that lysosomal RNF13 suppresses endo‐lysosome progression.

To specifically elaborate the effect of RNF13, we utilized *Rnf13* knocked out (*Rnf13* ^−/−^) mice that was a gift from professor Dahai Zhu, Peking Union Medical College.^[^ ^16^ ^]^ We prepared peritoneal macrophages from *Rnf13* ^+/+^ and *Rnf13* ^−/−^ mice, and found that *Rnf13* ^−/−^ macrophages had decreased mRNA and protein level of IL‐6 and IFN‐β than cells from *Rnf13* ^+/+^ mice in response to LPS, Poly I: C and CpG (Figure 1D; Figure S2C, Supporting Information). Moreover, cells overexpressed RNF13 treated with LPS, Poly I: C and CpG, respectively, expressed more IL‐6 and IFN‐β compared with control cells, both in mRNA and protein level (Figure 1E; Figure S2D, Supporting Information). Taken together, these data suggest an immune‐promoting effect of RNF13 in the response to endosomal TLRs activation in macrophages.

### 2.2 RNF13 Deficiency Protects Mice Against Endosomal TLRs Ligands and Bacterial Challenge

**Figure 2:**
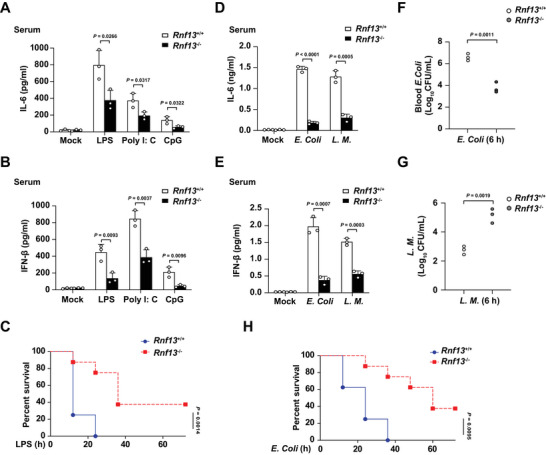
RNF13 deficiency protects mice against endosomal TLRs ligands and bacterial challenge. A) ELISA assay of IL‐6 in the serum of *Rnf13* ^+/+^ and *Rnf13* ^−/−^ mice infected with LPS (10 µg g^−1^), Poly I: C (100 µg g^−1^) and CpG (20 µg g^−1^) respectively for 2 h. B) ELISA assay of IFN‐β in the serum of *Rnf13* ^+/+^ and *Rnf13* ^−/−^ mice infected with LPS (10 µg g^−1^), Poly I: C (100 µg g^−1^) and CpG (20 µg g^−1^), respectively for 2 h. C) Survival curves of 6‐8‐week‐old mice of *Rnf13* ^+/+^ and *Rnf13* ^−/−^ mice infected with LPS (25 µg g^−1^) via intraperitoneal injection. D) ELISA assay of IL‐6 in the serum of *Rnf13* ^+/+^ and *Rnf13* ^−/−^ mice infected with *E. Coli* (1 × 10^6^ g^−1^) and *L. M*. (2 × 10^3^ g^−1^) respectively for 6 h. E) ELISA assay of IFN‐β in the serum of *Rnf13* ^+/+^ and *Rnf13* ^−/−^ mice infected with *E. Coli* (1 × 10^6^ g^−1^) and *L. M*. (2 × 10^3^ g^−1^) respectively for 6 h. F) *E. Coli* bacterial load in blood from *Rnf13* ^+/+^ and *Rnf13* ^−/−^ mice corresponding to Figure D and E. G) *L. M*. bacterial load in blood from *Rnf13* ^+/+^ and *Rnf13* ^−/−^ mice corresponding to Figure D and E. H) Survival curves of 6‐8‐week‐old mice of *Rnf13* ^+/+^ and *Rnf13* ^−/−^ mice infected with *E. Coli* (2.5 × 10^6^ g^−1^) via intraperitoneal injection. Data are shown as mean ± SD of n = 3 biological replicates (A, B, D–G) or demonstrated as Kaplan‐Meier survival curve (C, H, n = 8), two‐tailed unpaired Student's *t*‐test (A, B, D‐G) or Log‐rank (Mantel‐Cox) test (C, H).

We further investigated the importance of RNF13 in host defense against endosomal TLRs‐triggered innate immune responses. We challenged *Rnf13* ^−/−^ mice with LPS, Poly I: C and CpG, respectively, and found *Rnf13* ^−/−^ mice produced significantly less IL‐6 and IFN‐β in serum and organs (liver, spleen and lung) than *Rnf13* ^+/+^ mice in response to TLR ligands (**Figure** **2**A,B; Figure S3A,B, Supporting Information). Accordingly, *Rnf13* ^−/−^ mice had prolonged survival relative to that of *Rnf13* ^+/+^ mice after challenge with LPS (Figure 2C). To assess the role of RNF13 in the host innate response to infection against gram‐negative or gram‐positive bacteria, we intraperitoneally injected *Rnf13* ^−/−^ mice with *Escherichia coli* (*E. Coli*) and *L. M*., respectively. The production of proinflammatory cytokines (IL‐6 and IFN‐β) in the serum of *Rnf13* ^−/−^ mice was much lower than that in *Rnf13* ^+/+^ mice after injection of bacteria (Figure 2D,E). Moreover, *Rnf13* ^−/−^ mice had a lower *E. Coli* bacterial load in the blood than *Rnf13* ^+/+^ mice (Figure 2F), which is in accordance with reported study that proinflammatory cytokines promote the dissemination of *E. Coli*.^[^ ^17^ ^]^ Furthermore, *Rnf13* ^−/−^ mice had significantly higher *L. M*. bacteria in serum than control mice (Figure 2G). Correspondingly, the survival of *Rnf13* ^−/−^ mice after lethal challenge with *E. Coli* was also prolonged compared with *Rnf13* ^+/+^ mice (Figure 2H). These data indicate that deficiency of RNF13 can attenuate the inflammatory innate response of host to endosomal TLRs ligands and bacteria, and protect mice from hyperinflammation and organ injury.

### 2.3 RNF13 Enhances Endosomal TLRs Signaling Activity

**Figure 3:**
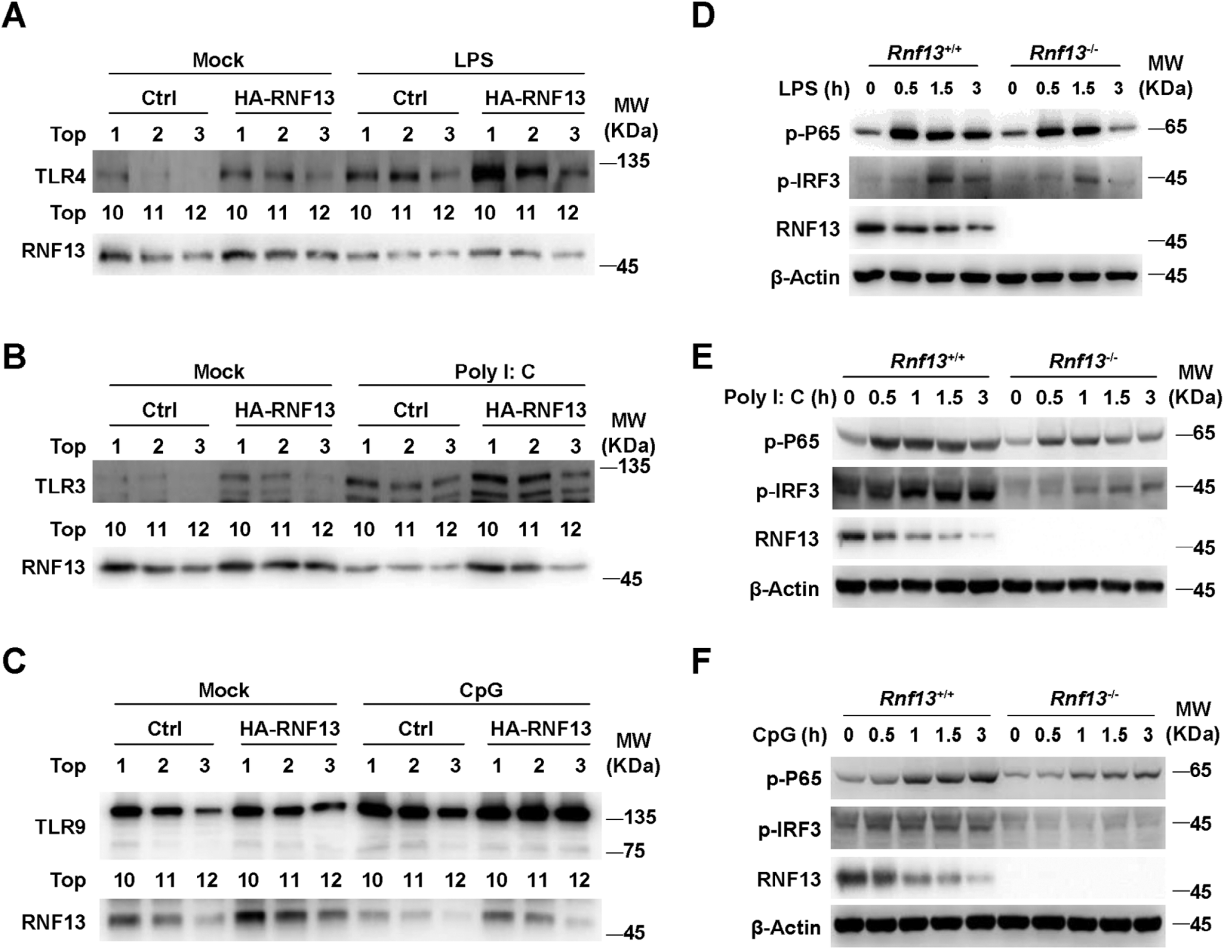
RNF13 enhances endosomal TLRs signaling activity. A) Western blot assay of Top1‐3 and Top10‐12 layers in subcellular fraction assay with control (Ctrl) and RNF13 overexpressing (HA‐RNF13) RAW264.7 cells treated with LPS (100 ng mL^−1^) for 0 or 3 h. B) Western blot assay of Top1‐3 and Top10‐12 layers in subcellular fraction assay with control (Ctrl) and RNF13 overexpressing (HA‐RNF13) RAW264.7 cells treated with Poly I: C (10 µg mL^−1^) for 0 or 3 h. C) Western blot assay of Top1‐3 and Top10‐12 layers in subcellular fraction assay with control (Ctrl) and RNF13 overexpressing (HA‐RNF13) RAW264.7 cells treated with CpG (2 µM) for 0 or 3 h. D) Western blot assay of TLR4 signaling pathway in *Rnf13* ^+/+^ and *Rnf13* ^−/−^ RAW264.7 cells treated with LPS (100 ng mL^−1^) for indicated hours. E) Western blot assay of TLR3 signaling pathway in *Rnf13* ^+/+^ and *Rnf13* ^−/−^ RAW264.7 cells treated with Poly I: C (10 µg mL^−1^) for indicated hours. F) Western blot assay of TLR9 signaling pathway in *Rnf13* ^+/+^ and *Rnf13* ^−/−^ RAW264.7 cells treated with CpG (2 µM) for indicated hours. Data are representative of three independent experiments (A–F).

We further investigated the effect of RNF13 on TLR‐activated downstream signal pathway in macrophages. We found the TLRs (TLR4, TLR3 and TLR9) in endosome fraction (Top 1–3 layer of subcellular fraction assay) were notably increased in RNF13‐ovexpressing cells with LPS, Poly I: C and CpG treatment, respectively (**Figure** **3**A–C). Consequently, deficiency of RNF13 resulted in less TLRs triggered phosphorylation of P65 (p‐P65) and IRF3 (p‐IRF3) in macrophages treated with LPS, Poly I: C and CpG, respectively (Figure 3D–F). Taken together, these data suggest that RNF13 promotes the endosomal TLRs level and TLR‐triggered signaling activity.

### 2.4 RNF13 Binds to LAMP‐1 and Decreases its Expression via RING Domain

To elucidate the mechanism how RNF13 regulates TLRs signaling, we identified RNF13‐interacting proteins by immunoprecipitation assay using an anti‐RNF13 antibody in mouse macrophages and followed with mass spectrometry analysis (IP‐MS). Our results revealed the conspicuous absence of TLRs protein among the potential RNF13‐interacting proteins (Table S1, Supporting Information). This remarkable revelation suggested that RNF13 may not act as a cofactor alongside TLRs to enhance TLR signaling.

**Figure 4:**
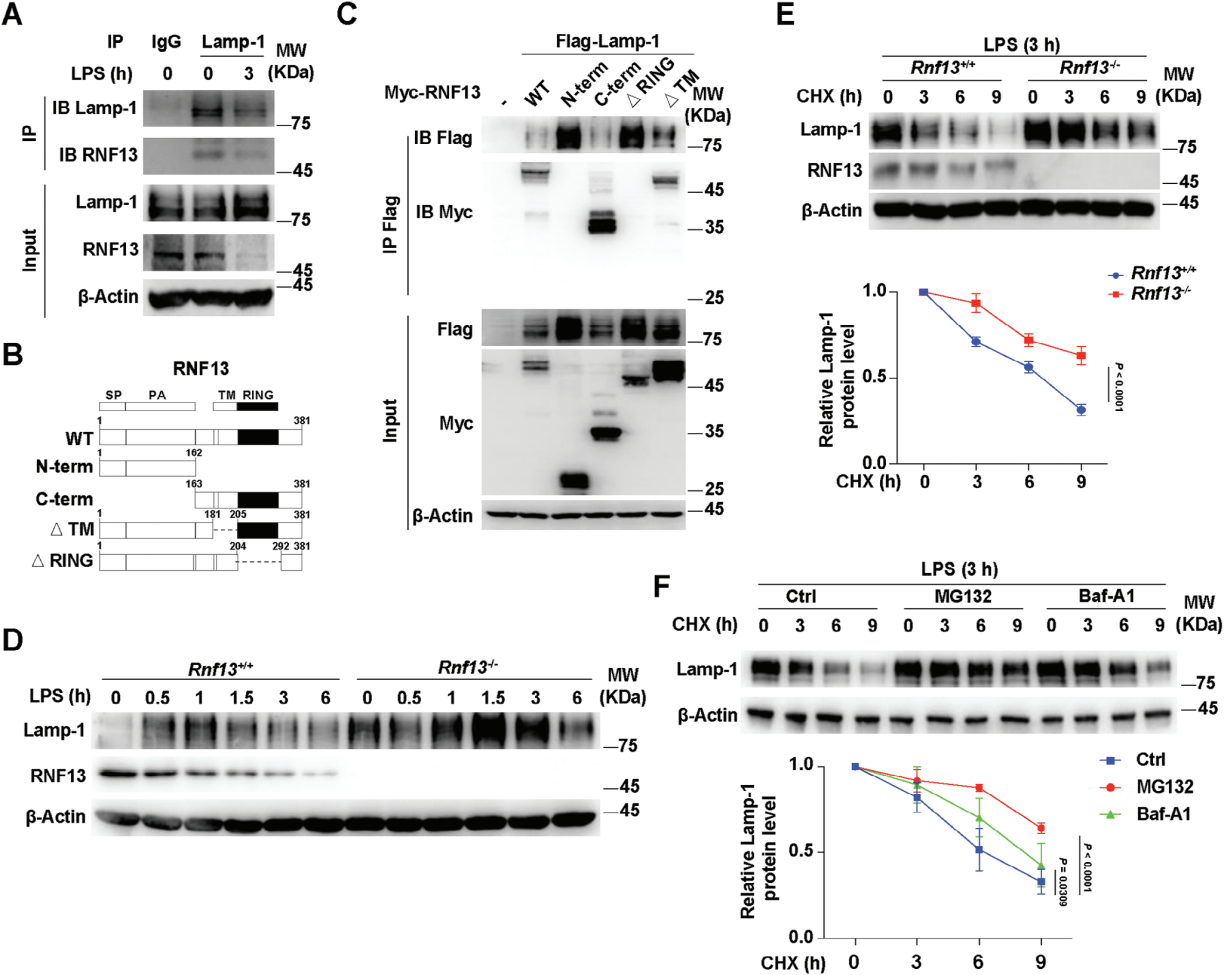
RNF13 binds to LAMP‐1 and decreases its expression via RING domain. A) Co‐immunoprecipitation analysis of endogenous interaction between RNF13 and LAMP‐1 in RAW264.7 cells along with LPS (100 ng mL^−1^) stimulation for indicated hours (IP: LAMP‐1). B) Schematic presentation of full‐length and truncations of RNF13. C) Co‐immunoprecipitation analysis of interaction between different truncations of RNF13 and LAMP‐1 in HEK293T cells transfected with different truncations of RNF13 and LAMP‐1. D) Western blot assay of LAMP‐1 protein level regulated by RNF13 in *Rnf13* ^+/+^ and *Rnf13* ^−/−^ RAW264.7 cells treated with LPS (100 ng mL^−1^) for indicated hours. E) Western blot analysis of RNF13's effect on protein level of LAMP‐1 in *Rnf13* ^+/+^ and *Rnf13* ^−/−^ RAW264.7 cells after LPS (100 ng mL^−1^) stimulation for 3 h and treated with CHX (100 µg mL^−1^) for indicated hours (Up). Relative expression level of LAMP‐1 is calculated by the ImageJ program (Down). F) Western blot analysis of MG132's and Baf‐A1's effect on protein level of LAMP‐1 in RAW264.7 cells after LPS (100 ng mL^−1^) stimulation for 3 h and treated with CHX (100 µg mL^−1^) for indicated hours (Up). Relative expression level of LAMP‐1 is calculated by the ImageJ program (Down). Data are representative of three independent experiments (A, C–F) or shown as mean ± SD of n = 3 biological replicates (E, F), two‐way ANOVA test (E, F).

We therefore investigated whether RNF13 interacts with endo‐lysosomal proteins and thus integrates into the TLRs signaling pathway. Among the RNF13‐interacting proteins, we identified LAMP‐1 that primarily localized to lysosomes (Table S1, Supporting Information). We then validated the binding between endogenous RNF13 and LAMP‐1 in the presence or absence of LPS treatment (**Figure** **4**A). In addition, by generating a variety of truncations of RNF13, we demonstrated that the RING domain of RNF13 played a critical role in their interaction (Figure 4B,C). Remarkably, we found that RNF13 deficiency significantly increased the protein level of LAMP‐1, independent of LPS stimulation (Figure 4D). Furthermore, it exerted a reduction in the rate of protein degradation of LAMP‐1 upon treatment with cycloheximide (CHX), an inhibitor of protein synthesis (Figure 4E). As LAMP‐1 itself is a lysosomal protein, we detected the effects of inhibitors of proteasomal (MG132) and lysosomal (Baf‐A1) degradation on LAMP‐1 expression, respectively, and found that the MG132 could sharply, while Baf‐A1 partially slowed down the CHX‐mediated decrease in the expression of LAMP‐1 (Figure 4F), indicating that degradation of LAMP‐1 is mainly mediated by proteasome and partially by lysosome pathway. These observations conclude that RNF13 effectively reduces the expression of LAMP‐1 by enhancing its proteasomal degradation, rather than inhibiting its synthesis. Taken together, our findings indicate that RNF13 binds to and degrades LAMP‐1.

### 2.5 RNF13 Induces LAMP‐1 Degradation by K48‐Linked Polyubiquitination

**Figure 5:**
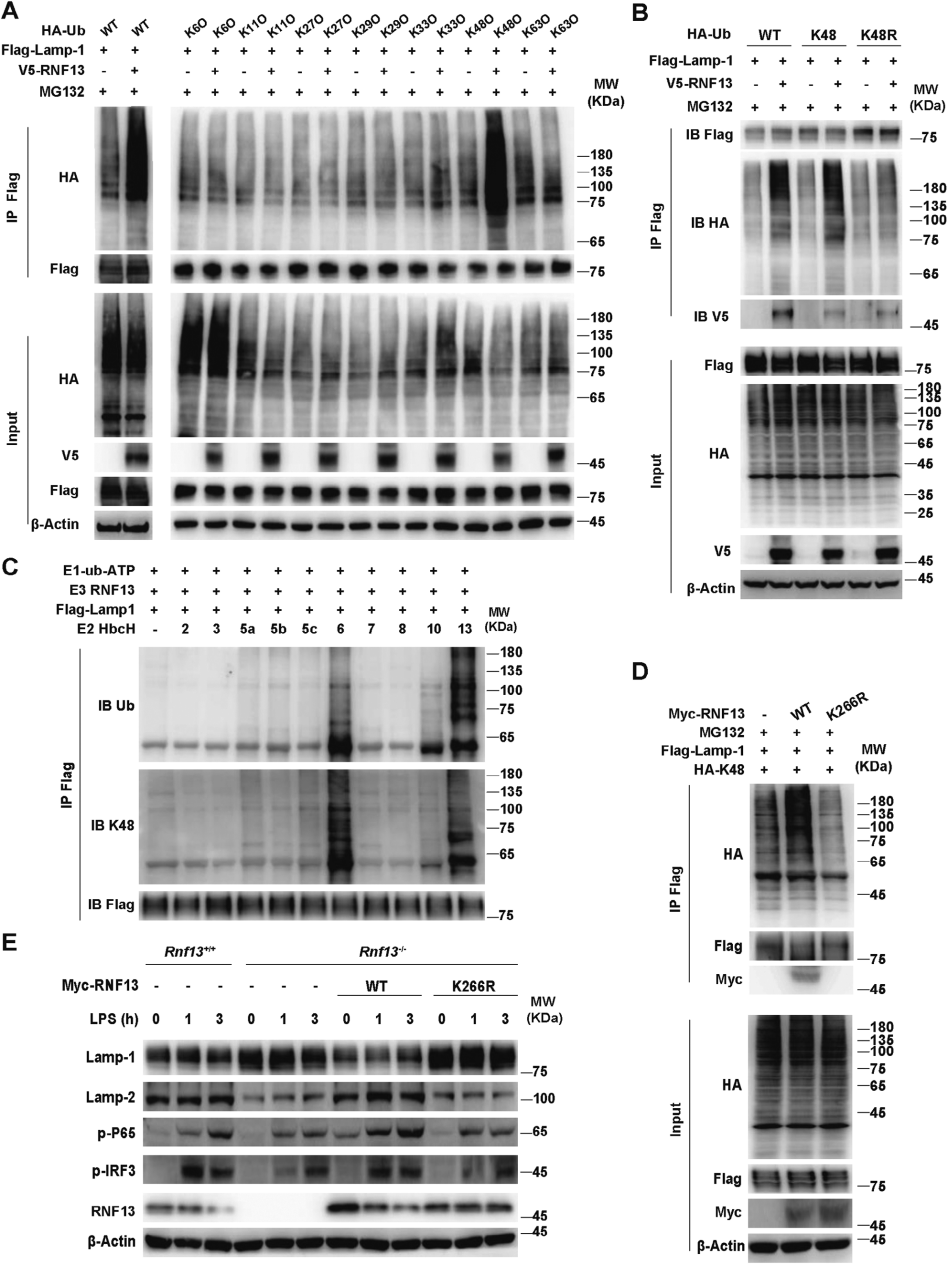
RNF13 induces LAMP‐1 degradation by K48‐linked polyubiquitination. A) Co‐immunoprecipitation analysis of ubiquitin type mediated by RNF13 on LAMP‐1 in HEK293T cells transfected with RNF13, LAMP‐1 and WT or K6/K11/K27/K29/K33/K48/K63 mutants of ubiquitin (Ub), and treated with MG132 (3 µM) for 5 h. B) Co‐immunoprecipitation analysis of ubiquitin type mediated by RNF13 on LAMP‐1 in HEK293T cells transfected with RNF13, LAMP‐1 and WT or K48/K48R of ubiquitin (Ub), and treated with MG132 (3 µM) for 5 h. C) In vitro ubiquitination assay of ubiquitination of LAMP‐1 mediated by RNF13 and related E2 enzymes (HbcH; numbers above lanes indicate that enzyme). D) Co‐immunoprecipitation analysis of K48‐linked polyubiquitination mediated by RNF13 (WT and K266R) on LAMP‐1 in HEK293T cells transfected with RNF13 (WT or K266R), LAMP‐1 and K48 of ubiquitin (Ub), and treated with MG132 (3 µM) for 5 h. E) Western blot assay of RNF13's effect on TLR4 signaling pathway in *Rnf13* ^+/+^, *Rnf13* ^−/−^ RAW264.7 cells and *Rnf13* ^−/−^ RAW264.7 cells overexpressed by WT or K266R of RNF13 respectively, treated with LPS (100 ng mL^−1^) for indicated hours. Data are representative of three independent experiments (A–E).

Then we investigate the type of polyubiquitin linkage that RNF13 mediated on LAMP‐1 by co‐transfecting different ubiquitin mutants (K6O, K11O, K27O, K29O, K33O, K48O and K63O, each of which had only one specific lysine valid for polyubiquitin linkage) with RNF13 and LAMP‐1 in HEK293T cells. We found that RNF13 significantly increased the K48‐linked polyubiquitination of LAMP‐1 among the different types of ubiquitin linkages compared to the RNF13‐untransfected control (**Figure** **5**A). Consistently, LAMP‐1ubiquitination mediated by RNF13 was abolished when K48 of ubiquitin was mutated to arginine (K48R) (Figure 5B). To determine whether RNF13 directly modified LAMP‐1, we reconstituted the LAMP‐1 ubiquitination reaction in vitro. We prepared various combinations of recombinant Flag‐tagged LAMP‐1, recombinant RNF13, purified E2 enzymes (HbcH2, HbcH3, HbcH5a, HbcH5b, HbcH5c, HbcH6, HbcH7, HbcH8, HbcH10 and HbcH13) and a mixture of E1 plus ubiquitin. We observed degradation and K48‐linked ubiquitination of LAMP‐1 in the presence of RNF13 and the E2 enzymes HbcH6 and HbcH13 (Figure 5C). Taken together, these data indicate that RNF13 directly induces ubiquitination of LAMP‐1 through K48‐mediated linkage.

Since the K266R single amino acid point mutation in the RING domain of RNF13 has been reported to impair its E3 ligase activity,^[^ ^15^ ^]^ we generated the RNF13K266R mutant and found that the K266R mutant did not increased the K48‐linked polyubiquitination of LAMP‐1 any more (Figure 5D). Furthermore, overexpression of the wild‐type (WT), but not the K266R mutant of RNF13 in *Rnf13* ^−/−^ macrophages reduced the expression of LAMP‐1 and increased the phosphorylation levels of P65 (p‐P65) and IRF3 (p‐IRF3) (Figure 5E), suggesting that RNF13 promotes the inflammatory response through ubiquitin‐mediated degradation of LAMP‐1. Notably, we found that the protein level of LAMP‐2 was negatively correlated with the expression of LAMP‐1 (Figure 5E), consistent with the idea that LAMP‐1 deficiency could be compensated by LAMP‐2 upregulation.^[^ ^18^ ^]^ Taken together, these data suggest that RNF13 mediates K48‐linked polyubiquitination of LAMP‐1 to promote its proteasomal degradation, thereby promoting the inflammatory response.

### 2.6 LAMP‐1 Suppresses TLR Signaling Pathway by Promoting the Progression of Lysosome

**Figure 6:**
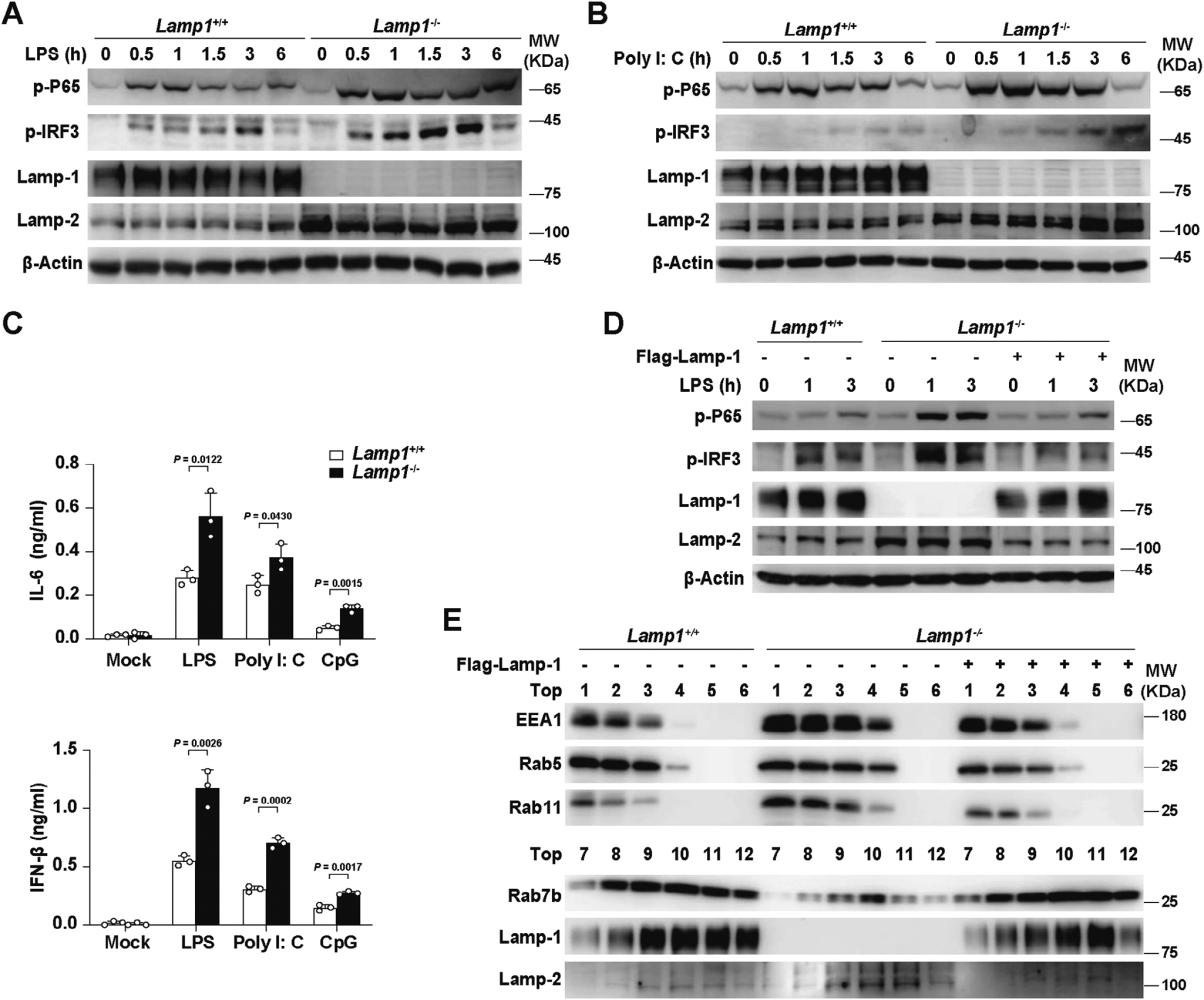
LAMP‐1 suppresses TLR signaling pathway by promoting the progression of lysosome. A) Western blot assay of LAMP‐1′s effect on TLR4 signaling pathway in *Lamp1* ^+/+^ and *Lamp1* ^−/−^ RAW264.7 cells treated with LPS (100 ng mL^−1^) for indicated hours. B) Western blot assay of LAMP‐1′s effect on TLR3 signaling pathway in *Lamp1* ^+/+^ and *Lamp1* ^−/−^ RAW264.7 cells treated with Poly I: C (10 µg mL^−1^) for indicated hours. C) ELISA assay of IL‐6 and IFN‐β in the supernatants of *Lamp1* ^+/+^ and *Lamp1* ^−/−^ RAW264.7 cells treated with LPS (100 ng mL^−1^), Poly I: C (10 µg mL^−1^) or CpG (2 µM) for 6 h respectively. D) Western blot assay of LAMP‐1′s effect on TLR4 signaling pathway in *Lamp1* ^+/+^, *Lamp1* ^−/−^ RAW264.7 cells and *Lamp1* ^−/−^ RAW264.7 cells overexpressed by LAMP‐1, treated with LPS (100 ng mL^−1^) for indicated hours. E) Western blot assay of LAMP‐1′s effect on subcellular fraction (Top1‐6) in *Lamp1* ^+/+^, *Lamp1* ^−/−^ RAW264.7 cells and *Lamp1* ^−/−^ RAW264.7 cells overexpressed by LAMP‐1, treated with LPS (100 ng mL^−1^) for 3 h. Data are representative of three independent experiments (A, B, D, E) or shown as mean ± SD of n = 3 biological replicates (C), two‐tailed unpaired Student's *t*‐test (C).

To identify the function of LAMP‐1 in TLRs‐triggered innate response, we generated *Lamp1* knocked out (*Lamp1* ^−/−^) RAW264.7 cells with CRISPR‐Cas9 system. Upon LPS or Poly I: C stimulation, TLRs‐mediated signaling activity was robustly enhanced in *Lamp1* ^−/−^ cells compared with that in wild‐type (*Lamp1* ^+/+^) cells (**Figure** **6**A,B). Accordingly, IL‐6 and IFN‐β were significantly increased in *Lamp1* ^−/−^ cells compared with that in *Lamp1* ^+/+^ cells upon LPS, Poly I: C or CpG stimulation (Figure 6C). Moreover, rescue of LAMP‐1 expression in *Lamp1* ^−/−^ cells could disturb TLR‐triggered signaling activity upon LPS treatment (Figure 6D). To clarify the function of LAMP‐2 in TLRs‐triggered inflammatory response, we knocked down the expression of LAMP‐2 in primary peritoneal macrophages upon LPS stimulation. We found that the level of LAMP‐1 was markedly increased and TLR4‐triggered pathway was robustly weakened in LAMP‐2 knockdown (si*Lamp2*) cells compared with those in control cells (si*NC*) (Figure S4A, Supporting Information). Notably, knockdown of LAMP‐2 had little effect on TLR4 signaling pathway in *Lamp1* ^−/−^ cells upon LPS treatment (Figure S4B, Supporting Information), indicating that LAMP‐2 inhibited TLRs signaling through LAMP‐1.

We next determine whether LAMP‐1 is involved in the progression of lysosome by performing subcellular fraction assay with *Lamp1* ^+/+^ and *Lamp1* ^−/−^ cells. We found that *Lamp1* ^−/−^ cells showed the increased protein level of endosome localized proteins (EEA1, Rab5 and Rab11) and reduced expression of lysosome localized proteins (Rab7b and LAMP‐1) compared with that in *Lamp1* ^+/+^ cells (Figure 6E). Along with that, the subcellular fractions containing endosome localized proteins were expanded in LAMP‐1 deficient cells, while those with lysosome localized proteins were shortened compared with that in control cells (Figure 6E). Moreover, these effect in *Lamp1* ^−/−^ cells was rescued by overexpressing LAMP‐1 (Figure 6E), indicating that LAMP‐1 promotes the progression of lysosome. Furthermore, immunofluorescence intensity of pHrodo was robustly weakened in *Lamp1* ^−/−^ cells upon LPS treatment, compared with that in control cells, which could be rescued by overexpressing LAMP‐1 in *Lamp1* ^−/−^ cells (Figure S4C, Supporting Information). Taken together, these data suggest that LAMP‐1 inhibits endosomal TLRs signaling pathway by promoting the progression of lysosome and degradation of TLRs.

### 2.7 RNF13 Induces LAMP‐1 Degradation by Mediating K48‐Linked Polyubiquitination on its K128 Position and is Involved in RA Pathogenesis

**Figure 7:**
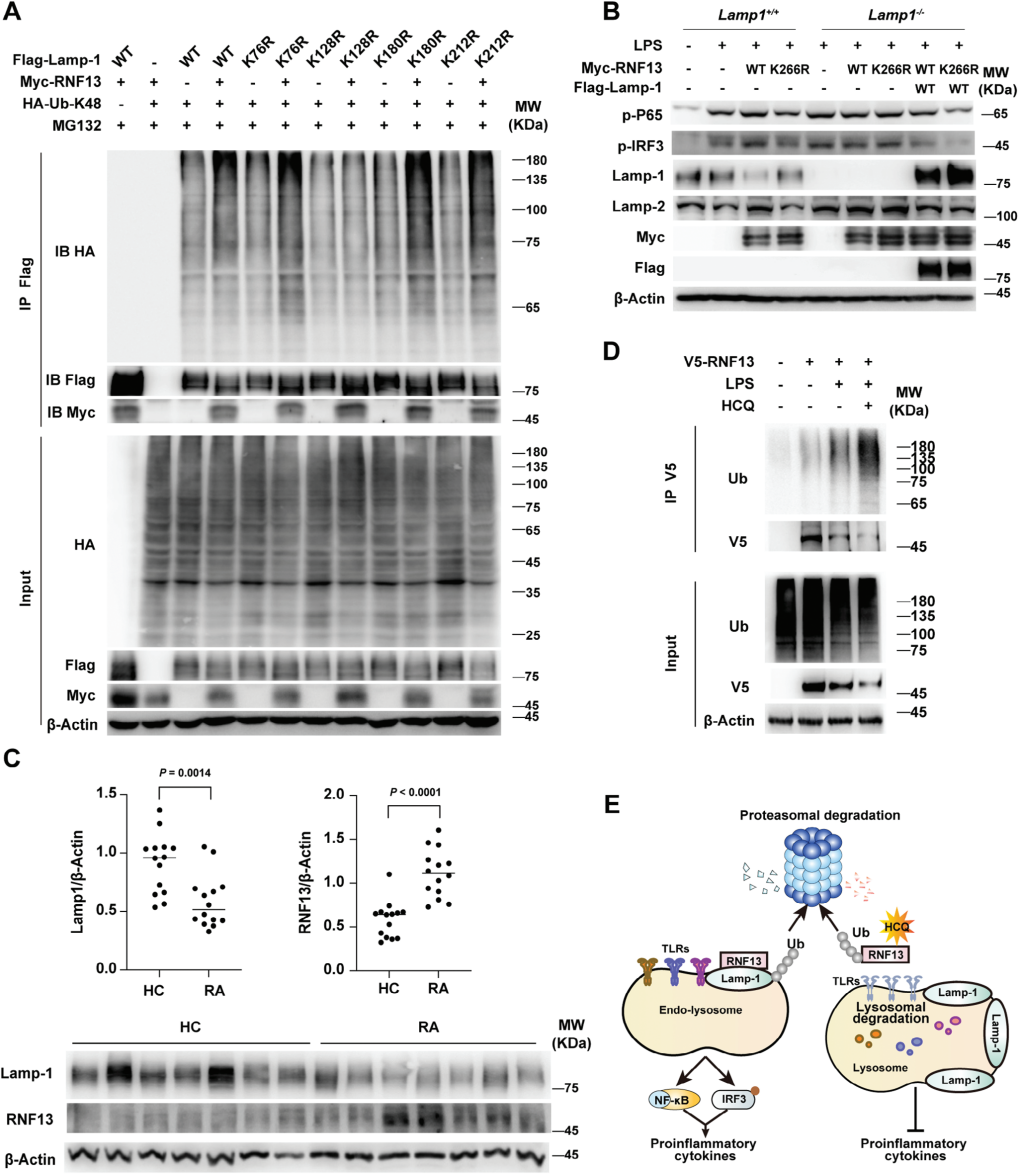
RNF13 induces LAMP‐1 degradation by mediating K48‐linked polyubiquitination on its K128 position and is involved in RA pathogenesis. A) Co‐immunoprecipitation analysis of K48‐linked polyubiquitination mediated by RNF13 on different LAMP‐1 point mutants in HEK293T cells transfected with RNF13, K48 ubiquitin (Ub) and different point mutants of LAMP‐1, and treated with MG132 (3 µM) for 5 h. B) Western blot assay of RNF13 (WT and K266R) and LAMP‐1′s effect on TLR4 signaling pathway in *Lamp1* ^+/+^, *Lamp1* ^−/−^ RAW264.7 cells and *Lamp1* ^−/−^ RAW264.7 cells overexpressed by LAMP‐1, transfected with WT or K266R of RNF13 and treated with or without LPS (100 ng mL^−1^) for 3 h. C) Relative intensity of RNF13 and LAMP‐1 in PBMCs from HC and RA patients that is calculated by the ImageJ program (Up). Western blot analysis of RNF13 and LAMP‐1 in PBMCs from HC and RA patients (Down). D) Co‐immunoprecipitation analysis of hydroxychloroquine's (HCQ) effect on polyubiquitination of RNF13 in THP‐1 cells transfected with V5‐RNF13 or not and treated with LPS (100 ng mL^−1^) for 3 h or not. E) Proposed working model for RNF13 as a positive regulator of endosomal TLRs‐mediated inflammatory response by targeting LAMP‐1 for K48‐linked polyubiquitination and degradation. Data are representative of three independent experiments (A, B, D) or shown as mean ± SD of n = 14 biological replicates (C), two‐tailed unpaired Student's *t*‐test (C).

To identify the ubiquitination sites of LAMP‐1 by RNF13, we generated different point mutants of LAMP‐1 (K76R, K128R, K180R and K212R) that were conserved among different species. We found that only K128R substitution blocked ubiquitination of LAMP‐1 via the K48‐mediated linkage by RNF13 (**Figure** **7**A). Additionally, RNF13, but not its K266R mutant, could enhance the TLR signaling activity upon LPS treatment when LAMP‐1 existed (Figure 7B). Taken together, these data indicate that RNF13 promotes the TLRs signaling pathway by mediating K48‐linked polyubiquitination at LAMP‐1 K128 site and consequent degradation.

Many autoimmune diseases (AIDs) are closely associated with hyperinflammatory responses.^[^ ^19^ ^]^ Rheumatoid arthritis (RA), a frequently occurring and deadly AID, is distinguished by chronic systemic inflammation.^[^ ^20^ ^]^ To investigate the clinical significance of RNF13‐LAMP‐1 molecular axis, we analyzed the protein level of RNF13 and LAMP‐1 in peripheral blood mononuclear cells (PBMCs) from healthy controls (HC) people and newly diagnosed RA patients. We found increased RNF13 and reduced LAMP‐1 expression in PBMCs from RA patients compared with that of HC (Figure 7C). These results indicate that abnormal expression of RNF13 and LAMP‐1 may be involved in the pathogenesis of inflammatory autoimmune disease. As a derivative of the heterocyclic aromatic compound quinoline, hydroxychloroquine (HCQ) has been used as antimalarial agents for many years. We examined whether HCQ affects the expression of RNF13 in the inflammatory response. Our findings reveal that HCQ significantly increases the ubiquitination and reduces the protein level of RNF13 upon LPS treatment (Figure 7D), consistent with its anti‐inflammatory effects.

## 3 Conclusion and Discussion

The progression of endo‐lysosome is essential for the activation of endo‐lysosome‐localized TLRs, because proper acidification of the endo‐lysosome is crucial for TLRs to recognize ligands and interact with adaptors.^[^ ^21^ ^]^ Furthermore, these events drive the active TLRs degradation via lysosome‐dependent pathway that is required to avoid excessive inflammatory responses.^[^ ^5^ ^]^ Minimal perturbation of the cascade of events results in abnormal inflammatory responses. In this study, we demonstrate that the E3 ubiquitin ligase RNF13 promotes the proteasomal degradation of LAMP‐1 in macrophages. Importantly, LAMP‐1 can promote the progression of lysosome maturation, enhancing endosomal TLRs trafficking to lysosome and causing increased TLRs degradation, leading to the suppression of TLR‐triggered production of proinflammatory cytokines and IFN‐I. And HCQ increases ubiquitination of RNF13 and its degradation by the proteasome, performing the anti‐inflammatory action (Figure 7E). Our study reveals a positive regulatory function of RNF13 in endosomal TLRs‐triggered signaling by promoting LAMP‐1 proteasomal degradation.

Lysosomal membranes contain several highly N‐glycosylated proteins, including LAMP‐1 and LAMP‐2. These two glycoproteins are structurally similar, evolutionarily related, and complement each other to a certain extent.^[^ ^22^ ^]^ However, they have different modes of assembly, which underlie their partially functional differences. For example, LAMP‐1 cannot compensate for the lack of LAMP‐2 in neutrophil phagocytes,^[^ ^23^ ^]^ and LAMP‐2 knockout mice have a high prevalence of periodontitis.^[^ ^24^ ^]^ It has been reported that LAMP‐2, but not LAMP‐1, is the substrate that undergoes K48‐linked ubiquitination by the CUL4A E3 ubiquitin ligase complex in damaged lysosomes.^[^ ^25^ ^]^ These results clearly showed that the LAMP proteins fulfil functions far beyond the initially suggested roles in maintaining lysosomal integrity and function. In the current study, we found that LAMP‐1‐deficient cells greatly reduced acidification and the fusion of late endosomes/lysosomes with autophagosomes in macrophages, and consequently, reduced trafficking of TLR4 to the degradation pathway and augmented the response to TLR agonists. In contrast, LAMP‐2 had little effect on inflammatory response. A recent study demonstrated that the presence of RNF13 L311S and L312P variants in lysosomal vesicles is diminished and affects the size of endosomal vesicles compared with RNF13 WT.^[^ ^26^ ^]^ Based on our findings, since RNF13 variants fail to interact with and degrade LAMP‐1, abnormal LAMP‐1 expression might accelerate the switch from early to late endocytic compartments, causing the enlargement of Lamp1‐positive vesicles. Other endosomal proteins, such as Rab family members Rab5 and Rab7, have also been shown to coordinate endosome maturation. Dysregulation of Rab5 or Rab7 is thought to disrupt endosome maturation, leading to enlarged endosomes and/or lysosomes.^[^ ^27^ ^]^ Thus, the challenge is to understand how these proteins coordinate to control the endosomal maturation and the properties of late endosomal and lysosomal compartments.

TLRs traverse the endosomal pathway to locate the ligand recognition sites and are eventually degraded.^[^ ^28^ ^]^ In our view, TLRs signaling accompanied by receptors loss aims to maintain TLRs homeostasis. Several studies have provided substantial evidence that dysregulation of endosomal TLR signaling contributes to the development and progression of autoimmune diseases such as RA and systemic lupus erythematosus (SLE).^[^ ^3^, ^29^ ^]^ HCQ is an immunomodulatory drug to treat malaria and autoimmune diseases.^[^ ^30^ ^]^ We found that increased RNF13 correlated with decreased expression of the substrate LAMP‐1 in RA patients, and that HCQ promoted RNF13 ubiquitination degradation, in line with its antirheumatic properties through interference with TLR processing in macrophages.

In this study, we report that RNF13 promotes the inflammatory response by combining LAMP‐1‐proteasomal degradation with TLR‐lysosomal degradation during the TLR‐triggered innate immune response. Future studies on the temporal and spatial regulatory mechanisms of the intracellular TLRs will provide a potential therapeutic target to inhibit or enhance the TLRs response.

## 4 Experimental Section

### RA Patients and Healthy Control Donors

All RA patients (age ranging from 29 to71, mainly female) fulfilled the American College of Rheumatology (ACR) classification criteria for RA.^[^ ^31^ ^]^ Patients with concurrent infection or other severe disorders were excluded from the study. Healthy control (HC) donors had no history of autoimmune diseases, had no inflammatory or infectious disease recently, and were age‐ and sex‐matched individuals for the RA patients. The study was approved by the Research Ethics Board of Peking Union Medical College Hospital (JS‐1196). Written informed consent was signed before sample collection. Information of healthy donors and RA patients were listed in Table S2 (Supporting Information).

### Mice

RNF13‐deficient mice based on C57BL/6J background were a gift from professor Dahai Zhu, Peking Union Medical College.^[^ ^16^ ^]^ Primary peritoneal macrophages were collected from wild‐type and *Rnf13* ^−/−^ mice 72 h after 3% thioglycolate intraperitoneal injection. All animal experiments were carried out according to the National Institutes of Health Guide for the Care and Use of Laboratory Animals, and with approval of the Scientific Investigation Board of Chinese Academy of Medical Sciences (ACUC‐A01‐2023‐030).

### Reagents and Antibodies

The CpG ODN, Poly I: C and lipopolysaccharide (LPS) were from Invivogen. LipofectamineLTX was from Invitrogen. FuGENE HD was from Promega. Lipofectamine RNAiMAX was from Invitrogen. The proteasome inhibitor MG132 and protein synthesis inhibitor cycloheximide (CHX) was from Sigma. The lysosome inhibitor Baf‐A1 was from Selleck. Antibodies used for immunoblot analysis were listed as follow: anti‐RNF13 was obtained from Abmart, anti‐LAMP‐1 (ab25245; Abcam), anti‐LAMP‐2 (ab37024; Abcam), anti‐TLR3 (NBP2‐24875; NOVUS), anti‐TLR4 (BS3489; Bioworld), anti‐P65 (8242S; CST), antibody to phosphorylation P65 (3033S; CST), anti‐IRF3 (4302S; CST), antibody to phosphorylation IRF3 (4947S; CST), anti‐EEA1 (3288S; CST), anti‐Rab5 (3547S; CST), anti‐Rab11 (5589P; CST), anti‐Rab7b (H00338382‐M01; Abnova), anti‐β‐Actin (M177‐3; MBL), anti‐HA (3724S/2999S; CST), anti‐Myc (14038S; CST), anti‐Flag (8146S; CST), anti‐V5 (13202S; CST). Anti‐HA, anti‐Myc, anti‐Flag and anti‐V5 beads were from Sigma.

### Cell Culture, Transfection and Stimulation

The HEK293T and RAW264.7 cell lines were purchased from American Type Culture Collection (ATCC) and were maintained in DMEM medium containing 10% FBS (Invitrogen‐Gibco). The HEK293T cells were transfected with LipofectamineLTX, and RAW264.7 cells were transfected by FuGENE HD.

Peritoneal macrophages were harvested from the peritoneal lavage fluid with DMEM by centrifugation (650 rpm, 5 min) and then were resuspended in DMEM containing 10% FBS. Peritoneal macrophages were transfected by Lipofectamine RNAiMAX.

Cells were stimulated with LPS (100 ng mL^−1^), CpG ODN (2 µm) and Poly I: C (10 µg mL^−1^). Then ELISA and Q‐PCR were taken to measure the concentration of IFN‐β and IL‐6 in culture supernatants and their mRNA level in cells, respectively.

### In Vivo E. Coli and L. M. Infection

6‐ to 8‐week‐old mice were infected with *E. Coli* (1 × 10^6^ g^−1^) and *L. M*. (2 × 10^3^ g^−1^) in 500 µL DMEM through intraperitoneally injection. Spleen, liver, and lung were isolated from mice 6 h after infection to extract RNA and then to measure the mRNA level of *Ifnb* and *Il6*. The concentration of IFN‐β and inflammatory cytokine in plasma were measured by ELISA. The survival of mice was supervised every 12 h for 60 h after intraperitoneally injection of *E. Coli* (2.5 × 10^6^ g^−1^) or LPS (25 µg g^−1^), respectively.

### Precipitation and Immunoblot Analysis

For RAW264.7 cells with or without stable expression of RNF13, lysates were incubated with anti‐HA Dynabeads or with protein A/G Dynabeads and anti‐LAMP‐1 antibody at 4 °C overnight. Then the bound complexes were separated by MagnaRack Magnetic Separation Rack and were washed to remove unspecific combination by wash buffer for four times. The bound complexes were boiled to break the combination and proteins were analyzed by immunoblot analysis with anti‐HA or anti‐LAMP‐1 antibody. For HEK293T cells transfected with expression plasmids encoding full‐length or truncations of Myc or V5‐tagged RNF13 or mutants of Flag‐tagged LAMP‐1, lysates were incubated with anti‐Myc, V5 or anti‐Flag Dynabeads at 4 °C overnight. The following procedures were similar to that of RAW264.7 cells.

### Ubiquitination

HEK293T cells were transfected with expression plasmids of Flag‐tagged full‐length LAMP‐1 or LAMP‐1 mutants with or without co‐expression of Myc‐tagged RNF13 and HA‐tagged ubiquitin. 24 h later, cells were treated with 3 µm MG132 for another 5 h and then were collected for immunoblot analysis directly or after immunoprecipitation with anti‐Flag Dynabeads.

### In Vitro Ubiquitination Assay

GST‐tagged RNF13 (100 ng) derived from *E. coli* and LAMP‐1 (100 ng) derived from HEK293T cells were incubated with E1 (100 ng), E2 (500 ng) and ubiquitin (2.5 µg) in 50 µL ubiquitination assay buffer containing 50 mm Tris‐HCl, 2.5 mm MgCl_2_, 0.5 mm DTT and 2 mm ATP. The total reaction system was incubated for 2 h at 30 °C. Then the interaction between RNF13 and LAMP‐1 was detected by immunoprecipitation and immunoblot analysis.

### Subcellular Fraction Assay

Ctrl, HA‐RNF13, *Lamp1* ^+/+^ and *Lamp1* ^−/−^ RAW264.7 cells were washed by PBS containing 1 mm MgCl_2_ and 0.9 mm CaCl_2_ for twice and common PBS once. Then cells were collected and resuspended with 10 mL homogenization buffer (20 mm Hepes pH 7.4, 150 mm NaCl, 2 mm CaCl_2_) and centrifuged at 4 °C, 6000 g for 10 min. The cells pellets were resuspended with threefold volume homogenization buffer and ruptured by pipetting up and down for 50 times. Cell lysis was centrifuged at 4 °C, 6000 g for 5 min twice. The final supernatant was carefully transferred to the top of 12 mL 20% percoll solution (10 mm Tris pH 8.0, 150 mm NaCl, 20% percoll) and centrifuged at 4 °C, 20 000 g for 1 h. Every 1 mL of the supernatant from the top was transferred to a new tube for 12 tubes. Then CHAPS was added to each tube with a final concentration of 10 mm. Samples were mixed briefly and then put on ice for 1 h, followed by centrifugation at 4 °C, 100 000 g for another 1 h. The supernatant was collected as different subcellular fractions for western blot analysis, named as Top1 to Top12, respectively.

### pHrodo Assay

*Rnf13* ^+/+^, *Rnf13* ^−/−^, *Lamp1* ^+/+^ and *Lamp1* ^−/−^ RAW264.7 cells were seeded on 96/12‐well plates as 1 × 10^4^/3 × 10^5^ cells per well in advance. After treated with LPS, the cells were washed with PBS and then incubated with the pHrodoTM dextran at a final concentration of 50 µg mL^−1^ at 37 °C for 10 min. Then the cells were washed with PBS, followed by image shot in dye‐free medium. pHrodoTM Green dextran possesses a pH‐sensitive fluorescence emission that increases in intensity with increasing acidity, which is essentially non‐fluorescent in the extracellular environment but upon internalization, the acidic environment of the endosomes/lysosomes elicits a bright green‐fluorescent signal from this dextran conjugate.

### Quantitative RT‐PCR

RNA was extracted by the RNAfast200 Kit (Fastagen) in accordance with the manufacturer's instructions. The ReverTra Ace qPCR RT Master Mix Kit (TOYOBO) was used to synthesis cDNA from the extracted RNA by reverse transcription. SYBR Green Realtime PCR Master Mix (TOYOBO) was used for quantitative PCR. Primers used for Q‐PCR were listed in Table S3 (Supporting Information).

### Statistical Analysis

Two‐tailed unpaired Student's *t*‐test was applied to analyze the statistical significance of data from two groups with GraphPad Prism Software. Mice survival curve data was demonstrated as Kaplan–Meier curves and analyzed with log‐rank (Mantel–Cox) test. *P* values less than 0.05 were regarded as statistically significant.

## Conflict of Interest

The authors declare no conflict of interest.

## Author Contributions

W.L. and Y.W. contributed equally to this work. M.J. and X.C. designed and supervised the research; W.L., Y.W., and S.L. performed the experiments; X.Z. provided the clinical samples. W.L., X.C., and M.J. analyzed data and wrote the paper.

## Supporting information

Supporting Information

## Data Availability

The data that support the findings of this study are available from the corresponding author upon reasonable request.
