# Supplementary material for: E3 Ubiquitin Ligase RNF13 Suppresses TLR Lysosomal Degradation by Promoting LAMP‐1 Proteasomal Degradation
Source: Adv Sci (Weinh). 2024 Jun 21;11(32):2309560. doi: 10.1002/advs.202309560 (PMC11348240; doi:10.1002/advs.202309560)
Supplement: Supplementary file 1 — Supporting Information [file ADVS-11-2309560-s001.docx]

**Supporting Information**

**E3 Ubiquitin Ligase RNF13 Suppresses TLR Lysosomal Degradation by Promoting LAMP-1 Proteasomal Degradation**

*Wei Liu, Yuyang Wang, Shuo Liu, Xuan Zhang, Xuetao Cao ^*^, Minghong Jiang* *^*^*

**Figure S1. RNF13 suppresses endo-lysosome acidification and decreases upon TLR signaling pathway activation due to self-ubiquitination and degradation.**

**A**) The screening results of 103 E3 ubiquitin ligases effects on regulating the acidification of endo-lysosome in primary peritoneal macrophages treated with LPS (100 ng/ml) for 3 hours. The mean fluorescence intensity (MFI) of pHrodo represents the acidity of endo-lysosome and the MFI in si*NC* was normalized as 1. **B**) qRT-PCR analysis of relative mRNA level of *Rnf13* in macrophages transfected with siRNA targeting negative control (si*NC*) and *Rnf13* (si*Rnf13*) respectively. **C**) Western blot assay of RNF13 protein level in murine immune cells (Up). Relative intensity of RNF13 of western blot analysis is calculated by the ImageJ program (Down). **D**) Western blot assay of RNF13 level in RAW264.7 cells along with LPS (100 ng/ml), Poly I: C (10 μg/ml), CpG (2 μM) and *L. M.* (MOI = 10) stimulation for indicated hours. **E**) Co-immunoprecipitation analysis of RNF13’s effect on polyubiquitination of itself in HEK293T cells transfected with V5-RNF13, HA-Ubiquitin (Ub) or not, and treated with MG132 (3 μM) for 5 hours or not.

Data are shown as mean of n=3 biological replicates (A) or shown as mean ± s.d. of n=3 biological replicates (B) or representative of three independent experiments (C-E), two-tailed unpaired Student’s *t*-test (B).

**Figure S2. RNF13 promotes TLR-****triggered pro-inflammatory cytokine production in macrophages.**

**A**) Western blot analysis of RNF13 protein level in *Rnf13^+/+^* and *Rnf13^-/-^* RAW264.7 cells. **B**) Western blot analysis of RNF13 protein level in control (Ctrl) and RNF13 overexpressing (HA-RNF13) RAW264.7 cells. **C**) qRT-PCR analysis of *Il6* and *Ifnb* mRNA level in primary peritoneal macrophages from *Rnf13^+/+^* or *Rnf13^-/-^* mice treated with LPS (100 ng/ml), Poly I: C (10 μg/ml) or CpG (2 μM) for 3 hours respectively. **D)** qRT-PCR analysis of *Il6* and *Ifnb* mRNA level in control (Ctrl) and RNF13 overexpressing (HA-RNF13) RAW264.7 cells treated with LPS (100 ng/ml), Poly I: C (10 μg/ml) or CpG (2 μM) for 3 hours respectively.

Data are representative of three independent experiments (A, B) or shown as mean ± s.d. of n=3 biological replicates (C, D), two-tailed unpaired Student’s *t*-test (C, D).

**Figure S3. RNF13 deficiency protects mice against endo-lysosomal TLRs ligands challenge.**

A) qRT-PCR analysis of *Il6* mRNA level in liver, spleen and lung from *Rnf13^+/+^* or *Rnf13^-/-^* mice intraperitoneally treated with LPS (10 μg/g), Poly I: C (100 μg/g) and CpG (20 μg/g) respectively for 2 hours. B) qRT-PCR analysis of *Ifnb* mRNA level in liver, spleen and lung from *Rnf13^+/+^* or *Rnf13^-/-^* mice intraperitoneally treated with LPS (10 μg/g), Poly I: C (100 μg/g) and CpG (20 μg/g) respectively for 2 hours.

Data are shown as mean ± s.d. of n=3 biological replicates (A, B), two-tailed unpaired Student’s *t*-test (A, B).

**Figure S4. Lamp-1, but not Lamp-2, suppresses TLR signaling pathway by promoting the progress of lysosome.**

**A**) Western blot analysis of Lamp-2’s effect on TLR4 signaling pathway in peritoneal macrophages transfected with siRNA targeting negative control (si*NC*) or *Lamp2* (si*Lamp2*) and treated with LPS (100 ng/ml) for indicated hours. **B**) Western blot analysis of Lamp-2’s effect on TLR4 signaling pathway in *Lamp1^-/-^* RAW264.7 cells transfected with siRNA targeting negative control (si*NC*) or *Lamp2* (si*Lamp2*) and treated with LPS (100 ng/ml) for indicated hours. **C**) Images shot by HCS from pHrodo assay of endo-lysosome acidification in *Lamp1^+/+^*, *Lamp1^-/-^* RAW264.7 cells and *Lamp1^-/-^* RAW264.7 cells overexpressed by Lamp-1 with LPS (100 ng/ml) stimulation for 3 hours. The fluorescence intensity represents the acidity. (Scale Bar, 5 μm.)

Data are representative of three independent experiments (A-C) or shown as mean ± s.d. of n=3 biological replicates (C) or, two-tailed unpaired Student’s *t*-test (C).

**Table S1. Candidates of RNF13-interacting proteins from IP-MS analysis.**

| **Gene Symbol** | **LPS** | | **Poly I: C** | | **CpG** | |
| --- | --- | --- | --- | --- | --- | --- |
|  | **Score** | **Unique Peptides** | **Score** | **Unique Peptides** | **Score** | **Unique Peptides** |
| Atp1a1 | 214 | 19 | 285 | 19 | 41 | 5 |
| Hnrnpu | 211 | 14 | 282 | 14 | 254 | 10 |
| Wwp2 | 73 | 4 | 73 | 4 | N/A | N/A |
| Eif3c | 61 | 3 | 20 | 2 | N/A | N/A |
| Ipo5 | 49 | 1 | 96 | 5 | N/A | N/A |
| Lamp1 | 46 | 4 | 60 | 4 | 37 | 1 |
| Eef2 | 42 | 3 | 144 | 8 | N/A | N/A |
| Asap2 | 41 | 4 | 43 | 5 | 28 | 2 |
| Cse1l | 41 | 3 | 51 | 4 | N/A | N/A |
| A2m | 35 | 1 | 32 | 1 | 64 | 5 |
| Ap2b1 | 33 | 1 | 34 | 1 | N/A | N/A |
| Fam107b | 31 | 6 | N/A | N/A | N/A | N/A |
| Agpat1 | 29 | 1 | N/A | N/A | 25 | 3 |
| Xpo1 | 27 | 1 | 38 | 1 | N/A | N/A |
| Ddx21 | 26 | 5 | 79 | 5 | N/A | N/A |
| Eif3b | 25 | 1 | 50 | 3 | N/A | N/A |
| Tfrc | 24 | 4 | N/A | N/A | N/A | N/A |
| Ipo7 | 22 | 1 | N/A | N/A | N/A | N/A |

Candidates of RNF13-interacting proteins from IP-MS analysis in macrophages treated with LPS (100 ng/ml), Poly I: C (10 μg/ml) or CpG (2 μM) for 3 hours respectively. N/A: not detected.

**Table S2. Information of healthy donors and RA patients.**

|  | **Gender (F for female and M for male)** | **Age** |
| --- | --- | --- |
| Healthy donors | M | 32 |
|  | M | 60 |
|  | M | 65 |
|  | F | 28 |
|  | F | 30 |
|  | F | 33 |
|  | F | 35 |
|  | F | 43 |
|  | F | 48 |
|  | F | 51 |
|  | F | 58 |
|  | F | 60 |
|  | F | 60 |
|  | F | 63 |
| RA patients | M | 36 |
|  | M | 62 |
|  | M | 71 |
|  | F | 29 |
|  | F | 36 |
|  | F | 38 |
|  | F | 44 |
|  | F | 47 |
|  | F | 51 |
|  | F | 54 |
|  | F | 58 |
|  | F | 64 |
|  | F | 65 |
|  | F | 65 |

**Table S3. Sequences for Q-PCR primers.**

| **Gene name (Mouse)** | **Forward (5’-3’)** | **Reverse (5’-3’)** |
| --- | --- | --- |
| *Ifnb* | ATGAGTGGTGGTTGCAGGC | TGACCTTTCAAATGCAGTAGATTCA |
| *Il6* | TAGTCCTTCCTACCCCAATTTCC | TTGGTCCTTAGCCACTCCTTC |
| *Lamp1* | CAGCACTCTTTGAGGTGAAAAAC | ACGATCTGAGAACCATTCGCA |
| *Lamp2* | TGTATTTGGCTAATGGCTCAGC | TATGGGCACAAGGAAGTTGTC |
| *Rnf13* | AAGTGTGTAGATCCCTGGCTAA | GTCCGAGTCACCTTGGGAAG |
| *Gapdh* | AGGTCGGTGTGAACGGATTTG | TGTAGACCATGTAGTTGAGGTCA |
